# Multispectral imaging for presymptomatic analysis of light leaf spot in oilseed rape

**DOI:** 10.1186/s13007-019-0389-9

**Published:** 2019-01-24

**Authors:** Charles Veys, Fokion Chatziavgerinos, Ali AlSuwaidi, James Hibbert, Mark Hansen, Gytis Bernotas, Melvyn Smith, Hujun Yin, Stephen Rolfe, Bruce Grieve

**Affiliations:** 1 e-Agri Sensors Centre, School of Electrical and Electronic Engineering, University of Manchester, Sackville Street, Manchester, M1 3BU UK; 2 Department of Animal and Plant Sciences, University of Sheffield, Western Bank, Sheffield, S10 2TN UK; 3 Centre for Machine Vision, University of the West of England, Coldharbour Lane, Bristol, BS16 1QY UK

**Keywords:** Disease detection, Light leaf spot, Oilseed rape, Multispectral, Preprocessing, Machine learning, Support vector machine, Novelty detection, Orientation effects, Photometric stereo

## Abstract

**Background:**

The use of spectral imaging within the plant phenotyping and breeding community has been increasing due its utility as a non-invasive diagnostic tool. However, there is a lack of imaging systems targeted specifically at plant science duties, resulting in low precision for canopy-scale measurements. This study trials a prototype multispectral system designed specifically for plant studies and looks at its use as an early detection system for visually asymptomatic disease phases, in this case *Pyrenopeziza brassicae* in *Brassica napus*. The analysis takes advantage of machine learning in the form of feature selection and novelty detection to facilitate the classification. An initial study into recording the morphology of the samples is also included to allow for further improvement to the system performance.

**Results:**

The proposed method was able to detect light leaf spot infection with 92% accuracy when imaging entire oilseed rape plants from above, 12 days after inoculation and 13 days before the appearance of visible symptoms. False colour mapping of spectral vegetation indices was used to quantify disease severity and its distribution within the plant canopy. In addition, the structure of the plant was recorded using photometric stereo, with the output influencing regions used for diagnosis. The shape of the plants was also recorded using photometric stereo, which allowed for reconstruction of the leaf angle and surface texture, although further work is needed to improve the fidelity due to uneven lighting distributions, to allow for reflectance compensation.

**Conclusions:**

The ability of active multispectral imaging has been demonstrated along with the improvement in time taken to detect light leaf spot at a high accuracy. The importance of capturing structural information is outlined, with its effect on reflectance and thus classification illustrated. The system could be used in plant breeding to enhance the selection of resistant cultivars, with its early and quantitative capability.

## Background

The development of precision agriculture has brought about huge advances in monitoring technologies that allow for quantifiable and early detection of plant stress factors [1, 2]. Despite these technological advances and continuous improvement in plant varieties, yield improvements in many crops have plateaued in recent years [3]. This has been blamed in part on ineffective crop management, due to a lack of reliable tools for in-situ monitoring and intervention in increasingly varying conditions [4].

Plant disease is a leading contributor to global crop losses [5]. The selection of disease resistant crop varieties plays a central role in negating this diminution; typically achieved by visual scoring of symptom severity. Imaging techniques have considerable potential to improve this process, by enabling quantification of disease severity and development rate. This would facilitate breeding for crops with quantitative resistance; with individual traits and associated genes each contributing a small improvement to plant performance but, in combination, providing effective and sustainable disease resistance [6].

Plant diseases result in physiological and morphological alterations, with plant pigmentations caused by pathogen interactions [7]. In foliar fungal disease, infection is quantified by estimating the infected area on the leaf surface; a major limitation is the low accuracy due to the subjective nature of visual assessment [8]. Molecular methods have been exploited in plant pathology to improve the accuracy of diagnosis with serological assays, such as enzyme-linked immunosorbent assay (ELISA), used extensively for detection of pathogens [9] and nucleic acid-based methods, such as quantative polymerase chain reaction, based on using nucleotide primers with high affinity to target sequences. Although molecular methods are relatively low cost, they have disadvantages, as the sample preparation is destructive and labour intensive, and producing specific antibodies can be inefficient with the presence of inhibitors reducing the sensitivity of nucleic acid-based methods [10, 11]. As pathogens often do not spread uniformly inside plants, destructive molecular methods can be non-diagnostic, especially at the asymptomatic stage [10]. Therefore, new methods are required for precise non-invasive, non-destructive and continuous diagnosis.

The light leaf spot (LLS) pathogen (*Pyrenopeziza brassicae*) is an important disease of winter oilseed rape (OSR) (*Brassica napus*) [12–14]. This versatile crop has multiple uses, and is responsible for £0.7 billion of the UK agricultural market [15], with LLS responsible for losses of £400 million, between 2012 and 2014, with 95% OSR crops affected [16]. The onset of infection can be subtle, due to the hemibiotrophic nature of the pathogen. This makes in-situ analysis almost impossible during the initial infection stages [14, 17], relying on the emergence of late stage visual symptoms. Thus, novel approaches would benefit plant breeders seeking to develop new resistant varieties as well as farmers where early detection in the field opens the possibility of early intervention by sparse selective fungicide application, improving the efficacy of chemical applications.

Multispectral imaging (MSI) collect light reflected from the leaf surface. The spectrum of the reflected light is governed by both physical and biochemical interactions with the leaf, processes that will change during disease infection [18, 19]. Images are collected at different wavelengths in the optical range (350–1000 nm), where each pixel in the image represented by a vector, known as the spectral signature. Analysis typically uses spectral information to study plant properties and conditions through spectral vegetation indices (SVIs), used commonly in remote sensing to describe vegetation health and density [20]. Several SVIs have been developed to identify and detect plant disease [21, 22].

Using photometric stereo (PS) for analysing plant tissue has attracted limited attention in the literature. Recent work such as [23] has highlighted the usefulness of the approach for extracting high resolution 3D imagery of leaves in order to aid vein extraction. It has been shown that different wavelengths used for light sources affect the validity of the assumption that the surface exhibits Lambertian reflectance. For example in [24], reconstructions of human faces were shown to be more accurate under NIR than visible. This is thought to be due to the sub-surface scattering of NIR, which penetrates further into the skin, leading to a more diffuse reflectance. However, there is a compromise in using NIR for improving overall 3D reconstruction in that surface detail is lost. Whether the same issue affects plant tissue is not clear and will be the focus of further work, but for the purposes of this study visible light is used in order to extract as much higher resolution information as possible.

This study aims to develop a MSI system for disease diagnosis. This has been applied in a controlled environment for the analysis of disease progression and has the potential, with further development, to be used in the field [25]. This MSI system uses narrowband light sources and a broadband silicon detector, in contrast to typical MSI and HSI systems where a broadband illumination source is used with a narrow band (typically diffraction grating based) detector. This selected spectral resolution allows the technology to be applied at a fraction of the cost of traditional systems, whilst improving signal-to-noise ratio (SNR) due to targeted reflectance peaks. The use of machine vision algorithms allows for early symptom detection (before symptoms can be detected by eye) allowing better quantification of disease progression with the potential, in field production scenarios, to allow earlier and hence more effective intervention. The main contributions of this work lie in (1) introducing a refined MSI device, (2) detection of a hemibiotrophic infection before visible symptoms appear and (3) improvements in classification performance using tailored machine learning techniques.

## Materials and methods

The aim of this study was to demonstrate the detection capabilities of MSI whilst investigating the effect of monitoring at canopy scape versus leaf scale. Detached leaf and entire plant assays were undertaken to provide information with samples at different angles as found within canopies versus the orthogonal orientation achieved using detached leaves and compare the time to diagnosis between the two conditions.

### Host: oilseed rape

OSR (*Brassica napus* L.) has been selected as the host for these trials as it is an important crop and its broad leaf nature is useful to demonstrate the potential of canopy-scale imaging. Seeds (*Department of Biology, University of York*) were placed in Petri dishes on wet filter paper for three days until germination, when the seedlings were transferred to M3 (Levington) compost in $80\times 80\times 90$ mm square pots. The plants were grown at $16\,{^{\circ }}$C with a photo-period of 12h light/12h dark at 75% humidity for two weeks prior to inoculation.

### Pathogen: light leaf spot

**Fig. 1:**
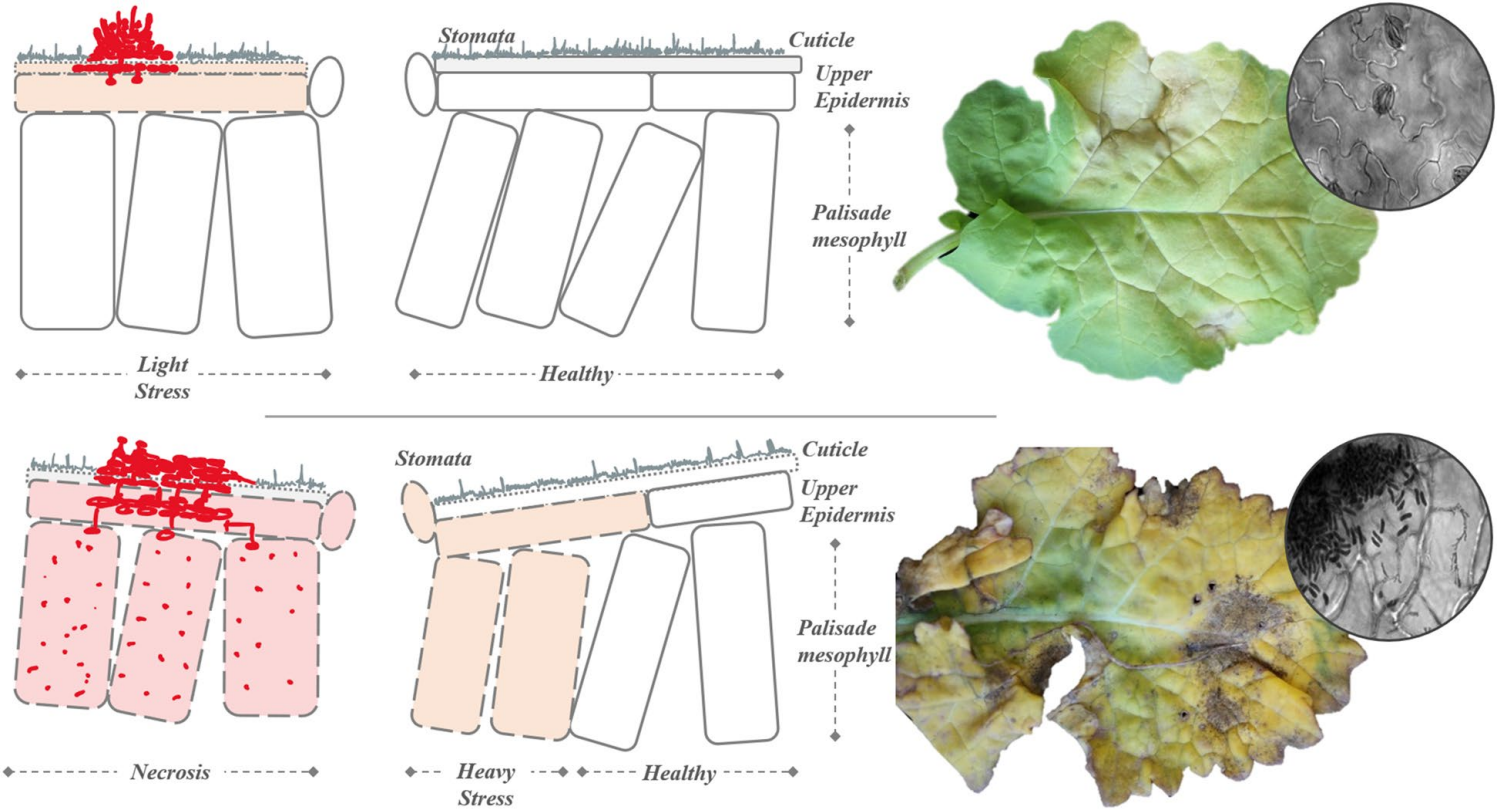
Pathogen life-cycle evolution. *Pyrenopeziza brassicae* infection necrotic development on leaf tissue showing early phase senescence (top) and lesions from a late-stage sample (bottom) both showing a colour-coded stage of infection. Included for information is a $\times$40 microscopy evaluation from each sample

*Pyrenopeziza brassicae* penetrates the cuticle directly as germ tubes [26], see Fig. 1, however no appressoria are formed and entry via stomata has not been confirmed [27]. The expression of cutinases assists the initial penetration of the cuticle. [26, 28]. *P. brassicae* has a hemibiotrophic lifestyle. The fungus initially grows as a hypomecylium where hyphae develop slowly in the subcuticular space between the cuticle and epidermal cells -no cell perforation or systematic spread is observed [27]. Therefore, the biotrophic phase of *P. brassicae* is visually symptomless, however the fungal growth will affect the plant tissue reflection properties [25], particularly in the NIR. Asexual sporulation signifies the later phase of infection when visual symptoms become apparent. The pathogen interacts with the metabolism of the host plants resulting in morphological and physiological perturbations such as stunting and chlorotic lesions, see Fig. 1. In common with many foliar pathogens, cytokinins are proposed to have a critical role in *P. brassicae* pathogenicity, promoting the formation of localised carbohydrate sinks and a redirection of nutrients from host to the pathogen. Sporulation causes the breaks in the cuticle and lesions develop, expanding concentrically. Chlorotic regions break down or become sunken due to the separation of the epidermal membrane and cuticular layer, or because of the production of toxins by the pathogen. In cases of severe infection, lesions merge and leaves become necrotic [17]. An LLS isolate (LLS160803, *Scotland’s Rural College*) was grown on Malt Agar Media (LabM) at $18\,{^{\circ }}\hbox {C}$ for two months. Conidia were collected by flooding the Petri dishes with 3–5 ml of sterile water plus 0.01% (v/v) Tween20 and agitating the mycelium with a plastic spreader. The spore suspension was collected in a 50ml Falcon tube and adjusted to $10^{6}$ spores/ml. The spore suspension was applied by spraying to plants when they had formed two true leaves. Infection was ensured by spraying the whole leaf surface area of the host until run-off. Mock controls were inoculated with sterile water plus Tween20 (0.01% v/v). The plants were covered immediately by plastic lids for 48h to maintain the leaf humidity above 90% and the temperature set at $16\,{^{\circ }}\hbox {C}$ [29].

### Trials

In order to investigate the pathogen-host interaction, alongside sensing limitations, two trials were used at different perspectives with plants and detached leaves.

#### Canopy assay

**Fig. 2:**
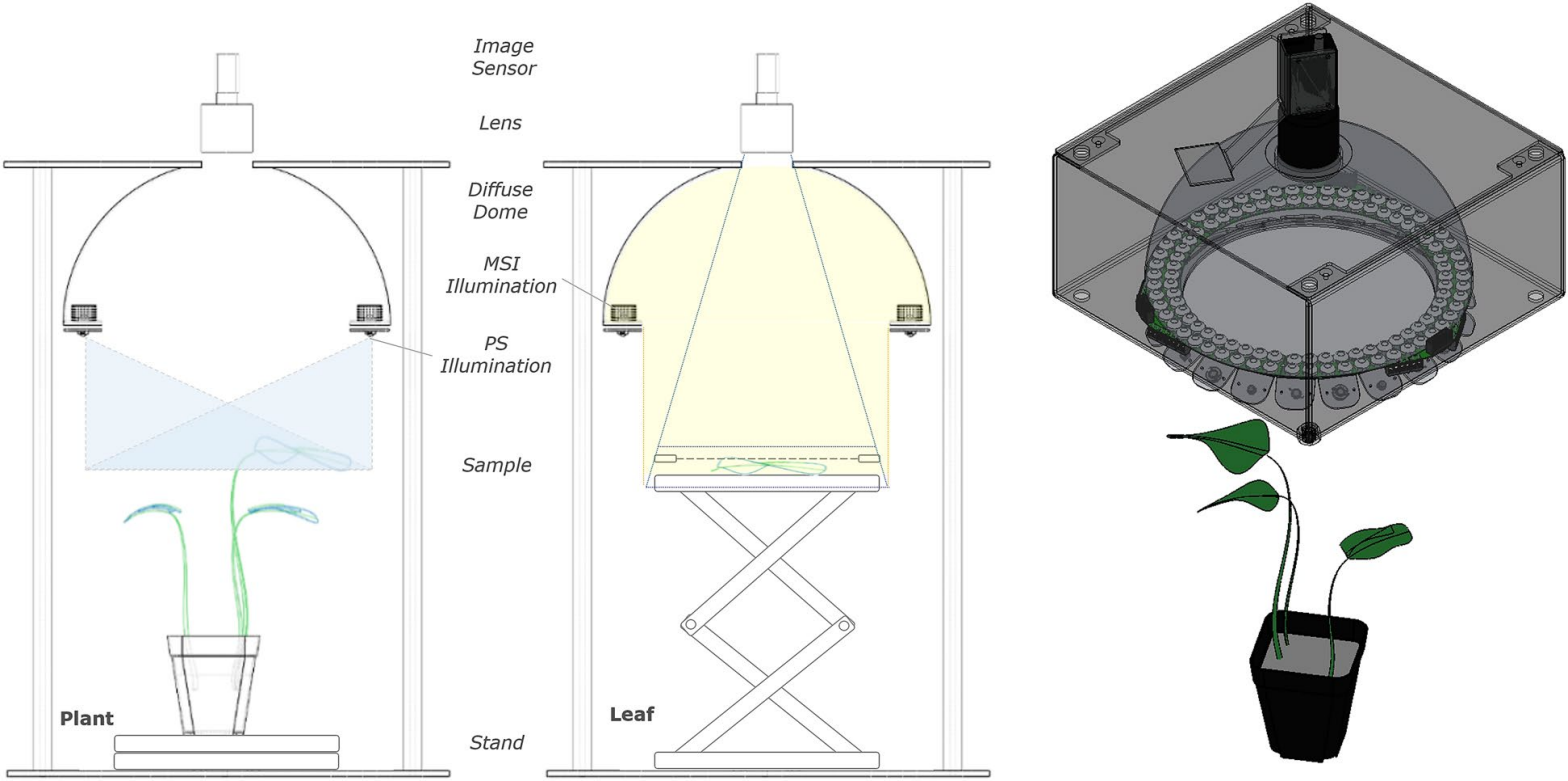
Scanning set-up. Side-view diagram of apparatus set-up for canopy and PS imaging (left) and detached leaf assay with MSI (centre) showing the major system components with three-dimensional view (right)

A susceptible OSR rape (*Brassica napus*) genotype, Charger, was selected based on moderate resistance rating (4/10) A [30]. The irradiance in the growth chamber was $200\;\upmu \hbox {mol m}^{-2}\hbox { s}^{-1}$. The study used 18 pathogen-inoculated replicates and 5 mock-inoculated with scans taken at 03, 06, 09, 12, 15, 18, 21, 24, 27 and 31 days after inoculation (DAI). Images were taken of the main canopy for all time points before 24 DAI and taken on a mounted leaf after, due to the size of the samples outgrowing the field of view, see Fig. 2.

#### Detached leaf assays

Four OSR genotypes, Bristol(2), Charger(4), Cracker(9), Temple(7), were selected on the basis of their resistance, as per their given ratings (/10) [30]. The irradiance in the growth chamber was $160\;\upmu \hbox {mol m}^{-2}\hbox { s}^{-1}$. At 25 DAI the inoculated leaves were removed with a sterile scalpel and placed on petri dishes within plastic trays with a small amount of distilled water. A small piece of tissue paper was wrapped around the leaf petiole and dipped into the water to keep the leaves moist. The study used 6 inoculated replicates and 6 mock-controls, from each of the four different genotypes. Data was collected at 26, 28, 31 and 34 DAI. Images were taken of leaf samples held in place using a wire mesh, see Fig. 2.

### Imaging equipment

A multispectral imaging prototype, see Fig. 2, developed at the e-Agri Sensors Centre, University of Manchester was used to obtain the data presented in this study. Detailed information on the system operation can be found in Veys et al. [25]. The system can be used with multiple NIR sensitive detectors; the ones used for this trial were an Omnivision 5647 and a Sony IMX219; both included a Bayer filter, whose response artefacts were removed via calibration [25]. The device contained 36 narrowband sources from 365–960 nm, full width half maximum (FWHM)  10 nm, illuminated sequentially. This transferred considerably less energy to the sample, whilst having better SNR, than the broadband illumination used in passive MSI imaging; this is important as the non-invasive nature of MSI is not realised if significant heat is transferred to the samples. The illumination was provided by back projecting lensed LEDs into a barium sulphate and latex [31] solution coated dome, which allowed homogeneous lighting of the sample. The current minimum spatial resolution is between 0.1 and 0.2 mm/pix at an object distance of 300 mm. Dark-field images were subtracted from the multispectral cube during the acquisition. The raw data were acquired onto an on-board USB-3 storage device during the trial, before being transferred to a host computer for analysis. The data are then normalised using a calibration file, created by using a barium sulphate white tile as a maximum target to prevent saturation of the image sensor and remove slight variation due to the LED intensity and non-linear camera response [25].

### Analysis software

The following sections include descriptions, with fundamental theory, on different processing and classification algorithms, which were written in MATLAB.

#### Background removal

The background was removed using an optimised soil adjusted vegetation index (OSAVI) mask, see Eq. (1) [32]:

1 $$\begin{aligned} OSAVI = (1+0.16)\frac{R800-R670}{R800+R670+0.16} \end{aligned}$$

where *Rxxx* parameters are the reflectance intensities at *xxx* nm and 0.16 the soil calibration factor. A mask is then corrected using an Otsu thresholding method [33]; a standard algorithm that finds the threshold that minimises the weighted sum of variances of the two classes in the histogram, below and above the threshold.

#### Region of interest identification

SVIs have long been linked to plant status and are particularly powerful for demonstrating spatial variation. This positional information was used to identify areas of infection to allow targeted reflectance values to be classified using machine learning techniques. The two SVIs of note in this study, for LLS detection were the Carter index 1 (CTR1) [34], used for its ability to detect plant stress as a ratio of red to violet, see Eq. (2):

2 $$\begin{aligned} CTR1 = \frac{R700}{R420} \end{aligned}$$

and the light leaf spot index (LLSI), developed during this trial to detect areas of LLS infected tissue, see Eq. (3):

3 $$\begin{aligned} LLSI = \frac{R720-R530}{R720+R530}-R830 \end{aligned}$$

where the Rxxx values have the same meaning as in Eq. (1). The index is based on the findings of the Cercospora leaf spot (CLS) index, which showed promise in a related analysis [35], with the values edited to target this pathogen/host variety based on maximising the variation within the SVI matrix. The reason the same index could not be used for both trials was due to the variance in reflected intensity in the NIR bands at different orientations. This meant that the LLSI worked much better with the flat samples while the CTR1 was less affected by sample angle. In order to identify regions of interest (ROI) the resulting SVI is thresholded again using the Otsu method [33]. Other SVIs used for classification comparison are referenced in Table 1. These were selected heuristically due to their spatial differentiation on the dataset and published links to disease-stress symptoms.

#### Data extraction

Spectral signatures were automatically extracted, using random indexing within the identified regions of interest of each replicate. For the analysis 690 pixels were extracted for entire plants, 150 control and 540 inoculated, and 435 pixels were extracted for individual leaves, 222 control and 213 inoculated. Each of these samples was labelled whether from an inoculated or control plant to aid classifier training and allow calculation of error. The number of samples extracted was increased until there was minimal variation present within each replicate set. The standard deviation within the sub-sampled information was negligible for this number of samples, which can been seen in the standard deviation in the classification.

#### Spectral processing

To aid the analysis, unwanted variation between wavebands is removed whilst the spectrum is preserved by using an appropriate Savitsky–Golay (SG) smoothing filter, see Eq. (4):

4 $$\begin{aligned} I_{SGj} = \sum _{i=-3}^{3}c_{i}I_{Ri+j} \end{aligned}$$

$I_{SG}$ is the smoothed intensity spectrum, $I_{R}$ is the raw intensity and *c* the convolution coefficients, calculated using a window size of seven and fifth order polynomial. Then, in order to compare the signature shape and negate a shift in the magnitude of the whole spectra due to slight varying of distances, a standard normal variate (SNV) normalisation is applied, to all wavelengths of a pixel, see Eq. (5):

5 $$\begin{aligned} z_{ip} = \frac{x_{ip} - \bar{x_{p}} }{s_{p}} \end{aligned}$$

where $z_{ip}$ is the processed spectra, and:

6 $$\begin{aligned} \bar{x_{p}} = \sum _{i=1}^{N} \frac{x_{ip}}{N} \end{aligned}$$

and

7 $$\begin{aligned} s_{p} = \frac{ \sum _{i=1}^{N}(x_{ip}-\bar{x_{p}})^{2}}{N-1} \end{aligned}$$

These remove a large proportion of noise variation and help to mitigate the scattering artefacts of reflectance imaging. Without the pre-processing steps, then the classification algorithms will start to differentiate the variance due to unwanted artefacts such as distance from camera variations in leaf angle.

#### Data redundancy reduction

In order to minimise the processing time, feature selection (FS) was used. FS is the process of removing redundant features and retaining those relevant to the problem that is being investigated [36]. Finding the informative subspace not only leads to a better utilisation of data storage, but also improves the predictive performance. A correlation-based feature selection (CFS) algorithm [37] was applied to the MSI signatures, to remove wavelengths that were not informative for disease classification. The concepts of information theory, Shannon’s entropy *H*(*x*), see Eq. (8):

8 $$\begin{aligned} H(x)=-\sum _{j=1}^{m}{P(x_{j})\log _2{P(x_{j})}} \end{aligned}$$

where $P(x_{j}) = Pr[X_{i} = x_{i}]$, and information gain $I({\varvec{x}},{\varvec{y}})$, see Eq. (9):

9 $$\begin{aligned} I({\varvec{x}},{\varvec{y}})=H(x)-H(x|y) \end{aligned}$$

were used in CFS to measure average feature-class $\bar{r_{cf}}$ and feature-feature $\bar{r_{ff}}$ correlations since it minimises the information gain bias introduced to the features and normalises the values [37]. The wavelengths were then evaluated heuristically to determine the most significant when considering LLS investigations, see Eq. (10):

10 $$\begin{aligned} \hbox {Merit}_{\mathrm{s}}=\frac{\hbox {N}\bar{\hbox {r}_{\mathrm{cf}}}}{\hbox {N}+\left( \hbox {N}+\hbox {N}\left( \hbox {N-1}\right) \right) \bar{\hbox {r}_{\mathrm{ff}}}} \end{aligned}$$

*N* is the number of wavelengths. The search was based on first-best and the process terminated if no improvement was achieved after five consecutive runs.

#### Conventional classification

The support vector machine (SVM) was employed in this study. Samples were grouped based on scale and variety, due to varying resistance, across current time series and preceding dates; to prevent a new algorithm for each time point. It was developed for two-class classification problem, in which the optimal hyperplane, defined as the maximal margin between the two classes, is used to classify the unseen test examples [38]. A training set is used to solve a quadratic problem for the best linear hyperplane, see Eq. (11):

11 $$\begin{aligned} \min _{\omega \in R^d, \xi _i\in R^+}\frac{\Vert \omega \Vert ^2}{2}+C\sum ^N_i\xi _i \end{aligned}$$

Subject to: $1-\xi _i \le y_i(\omega .\varvec{x_i}+b)$. Where $\omega$ represents the weight vector, *b* the learning bias, $\varvec{x_i}$ the training set, $\xi _i$ a non-zero slack variable, $y_i$ the desired class label, and *C* the regularisation parameter. Note that the regularisation parameter is used to penalise the misclassified samples, thus determining the flexibility of the decision boundary. Moreover, a radial basis function (RBF) kernel is often used to utilise the non-linear hyperplane, see Eq. (12):

12 $$\begin{aligned} K({\varvec{x}},{\varvec{y}}) = e^{-\gamma \Vert {\varvec{x}}-{\varvec{y}} \Vert ^2}, \quad \gamma =\dfrac{1}{2\sigma ^2} \end{aligned}$$

Half of the spectral signature samples were used for training with 10-fold cross-validation and the remaining samples were used for testing. The best parameters of the RBF kernel were achieved via cross-validation step (10-fold). The last step was substituting the weighting vector and the bias to solve the decision function, see Eq. (13):

13 $$\begin{aligned} f(x)=\mathrm {sgn}(\omega .K(\varvec{x_{i}},{\varvec{x}})-b) \end{aligned}$$

The value of decision function $f(x)\in {\pm 1}$ in which 1 denotes one class and $-1$ represents the other class. The classification rate was represented as an average of 100 independent runs, showing the number of times a pixel was misclassified as inoculated.

#### One-class classification

One-class SVM is an extension of the conventional SVM and it is used in unbalanced data cases. The lack of infected classes is usually due to the difficulties of obtaining them or the low frequencies of their detections. The model is generated to define the normal class boundaries, thus describing the unseen testing samples based on the boundaries. The output of the decision function was then further calibrated into class probability using non-decreasing (i.e. isotonic) regression [39]. Isotonic regression is an intermediary approach between binning and sigmoid fitting and it is achieved by employing a pair-adjacent violator routine to sort the training samples. This step is used to map the output onto the range of [0, 1] to define the best threshold as well as the classifier parameters. The calibrated output of the classifier, termed as ND-SVM in this study, is given as a score and used to differentiate the abnormal from the normal class, see Eq. (14):

14 $$\begin{aligned} z({\varvec{x}})=b-\omega .\phi ({\varvec{x}}_i) \end{aligned}$$

$\phi ({\varvec{x}}_i)$ represents the transformed vector ${\varvec{x}}_i$. This allows for automated limits to be set, depending on the training set, labelling all samples with FS wavelengths that lie outside the defined deviation to be classified as infected. It should be noted that if the training set includes control plants with other deficiencies (e.g. nutrient stress) then those conditions will be included in the accepted negative result classification.

### 3D reconstruction of plants

The 3D reconstruction of each sample was obtained using a PS facility integrated into the MSI system. This approach uses a circular array of point sources, in this case LEDs, positioned concentrically to the camera, then an image taken with each source illuminated in turn. This creates a series of images with differing illumination perspectives, allowing for the extraction of surface vectors. This is done by comparing the sample series of images with a calibration set of lighting vectors [24, 40], see Eq. (15):

15 $$\begin{aligned} {\mathbf {N}}(x, y) = \frac{{\mathbf {g}}(x,y)}{\mid \mid {\mathbf {g}}(x,y)\mid \mid } \end{aligned}$$

*N*(*x*, *y*) are the surface normal vectors, ${\mathbf {g}}(x, y)$ is defined in Eq. (16):

16 $$\begin{aligned} {\mathbf {g}}(x, y) = ( {\mathcal {L}}^{T}{\mathcal {L}})^{-1}{\mathcal {L}}^{T}\cdot {\mathcal {I}} \end{aligned}$$

${\mathcal {L}}$ is the illumination direction and ${\mathcal {I}}(x, y)$ is the backscattered reflectance intensity. This yields the surface normals, from which there are a number of methods to recover depth information. The Frankot–Chellappa algorithm [41] enforces integrability in Brooks and Horn’s algorithm [42] in order to recover integrable surfaces. Integrable surfaces are the ones that obey the relationship outlined in Eq. (17):

17 $$\begin{aligned} \frac{\delta {^2}f}{\delta _{x} \delta _{y}} = \frac{\delta {^2}f}{\delta _{y} \delta _{x}} \end{aligned}$$

This algorithm reconstructs the surface *f* by projecting *p*, *q* (the gradient fields) onto the set of integrable Fourier basis functions. Let $F(f(x,y))=\int \int f(x,y)e^{-j(\xi _{x}x+\xi _{y}y)}dxdy$ denote the Fourier transform of *f*(*x*, *y*). Thus, given *p*, *q*, then *f* is defined in Eq. (18):

18 $$\begin{aligned} f=F^{-1}\left( -j\frac{\xi _{x}F(p)+\xi _{y}F(q)}{\xi ^{2}_{x}+\xi ^2_y}\right) \end{aligned}$$

There are many other methods for reconstructing the surface from normals such as using the Discrete Cosine Transform to enforce integrability instead of the Fourier basis [43] or the M-estimator approach of [44], but the Frankot–Challappa method was chosen due to its efficiency and proven performance.

## Results

The system was used to analyse disease development in detached leaves and canopy. It was possible to detect infection using the MSI device before it became detectable via manual inspection methods. Canopy-scale analysis detected infected samples with a 92% (Charger) accuracy at 12 DAI, whilst the individual leaf-scale analysis had 83% (Bristol), 51% (Charger), 82% (Cracker), 83% (Temple) accuracies at 26 DAI respectively, see Table 4. Visible symptoms become apparent after 24 and 31 DAI for canopy and detached leaf trials respectively, although this varied slightly by cultivar. SVIs were used to quantify disease severity and provide a visual representation of disease distribution within the leaf or plant canopy.

**Table 1:** Average classification rate of LLS in OSR (Charger) using spectral indices and selected wavelength at canopy and leaf scale

Input	Average classification rate % (std)	
	Plant	Leaf
NDVI [45]	36.6 (0.02)	63.1 (0.02)
PSRI [46]	40.5 (0.04)	61.9 (0.02)
DWSI [47]	49.0 (0.04)	55.1 (0.02)
CLS [22]	52.2 (0.06)	65.6 (0.03)
CTR1 [34]	52.4 (0.05)	67.9 (0.03)
ARI [48]	56.8 (0.05)	70.5 (0.02)
PRI [49]	59.5 (0.04)	55.3 (0.02)
LLSI [this study]	59.5 (0.04)	75.0 (0.02)
MSI spectra	60.4 (0.05)	71.6 (0.02)
FS spectra	62.4 (0.05)	75.3 (0.02)

Note this classification uses one variety to compare the trials over all time points

### Multispectral backscattered reflectance

**Fig. 3:**
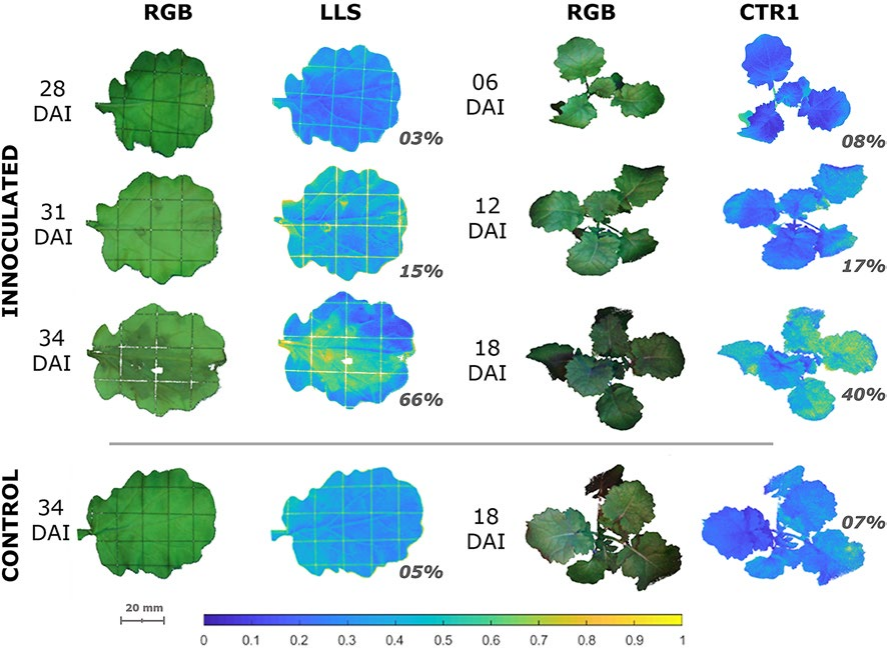
Infection Detection. Pathogen ingress detection demonstrated using the LLS for leaf and CTR1 for plant to outline disease severity, shown as a percentage, and distribution across a representative subset of the trial dates. The SVI colormap is normalised between 0 and 1 to display the infection intensity and distribution. The spatial scale is 1:25 with the 10 mm grid shown

**Fig. 4:**
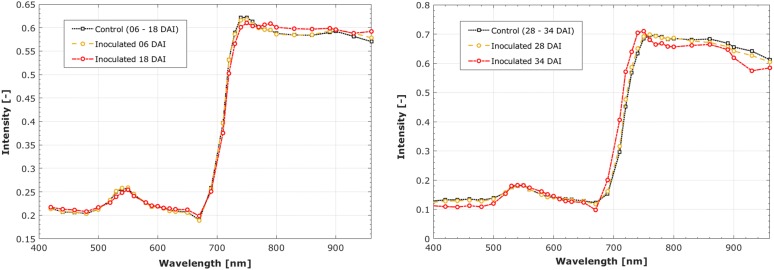
Reflectance Spectra. Reflectance spectra of ROI, identified by LLSI for detached leaves (left) and CTR1 for entire plants (right) normalised to a control spectra at each time-point. The FS wavelengths are highlighted to show points of differentiation

Due to the localised nature of the infection symptoms, the mean spectra of all plant tissue did not change significantly compared to the control. This is not surprising with early infection only present on 15% of the individual leaf surface, and inoculated leaves becoming occluded as the plant grows. However, by using thresholded SVIs to identify ROI (see Fig. 3) and then extracting spectra from these regions, the spectral changes due to infection can be seen, see Fig. 4.

### Support vector machine classification

The performance of SVIs, full MSI spectra and feature selection (FS) values for a conventional SVM classification were compared for both canopy and detached leaves within the same OSR variety (Charger). The FS outperformed the other approaches with values of 62% and 75% for plant and leaf images respectively, see Table 1. This is mainly due to the removal of superfluous information that provides misinformation to the classifier. The selected wavelengths may be of use for future experiments in order to reduce the acquisition and processing time whilst improving accuracy. The major wavelengths identified for this experiment were 520, 540, 580, 610, 630, 650 and 770 nm, these can be seen in Fig. 4. These wavelengths are consistent with the key values used in plant analysis, with areas inferring chlorophyll, carotenoid and phytochorome selected. These are of particular interest to the research community looking to develop portable devices with a cut-down number of wavelengths, targeted at particular duties.

**Table 2:** Classification rate improvement of using ND-SVM compared to conventional SVM for FS spectra of LLS in OSR entire plants (Charger)

DAI	Average classification rate % (std)	
	SVM	ND-SVM
03	61.6 (0.04)	82.6 (0.07)
06	64.4 (0.04)	83.4 (0.03)
09	66.7 (0.04)	88.3 (0.02)
12	73.3 (0.03)	92.2 (0.01)
15	75.0 (0.03)	92.3 (0.02)
21	78.8 (0.04)	93.5 (0.01)
24	91.8 (0.02)	94.5 (0.02)
27	94.2 (0.03)	94.7 (0.02)
31	97.7 (0.03)	96.7 (0.03)

**Table 3:** Classification rate comparison using conventional SVM for each cultivar with the resistance rating (/10) shown on fixed leaf data

DAI	Average classification rate % (std)			
	Bristol (2)	Charger (4)	Cracker (9)	Temple (7)
26	69.3 (0.03)	72.9 (0.05)	57.1 (0.06)	73.7 (0.06)
28	68.0 (0.04)	75.2 (0.06)	60.6 (0.06)	69.3 (0.07)
31	70.0 (0.04)	77.8 (0.08)	67.6 (0.09)	70.2 (0.08)
34	74.2 (0.06)	78.5 (0.04)	75.8 (0.11)	77.0 (0.09)

**Table 4:** Classification rate comparison using ND-SVM for each cultivar with the resistance rating (/10) shown on fixed leaf data

DAI	Average classification rate % (std)			
	Bristol (2)	Charger (4)	Cracker (9)	Temple (7)
26	82.8 (0.04)	50.8 (0.07)	81.8 (0.04)	82.9 (0.05)
28	81.7 (0.05)	61.8 (0.08)	82.5 (0.05)	74.0 (0.07)
31	85.3 (0.07)	77.2 (0.07)	80.6 (0.06)	81.2 (0.06)
34	87.0 (0.10)	80.7 (0.12)	83.8 (0.08)	87.0 (0.11)

### Novelty detection

Due to the localised nature of infection, the amount of data given to the SVM for infected tissue, despite ROI was limited, which constrained classification performance (see Table 1). Thus novelty detection (ND) was applied, using control plants for training and looking for instances that fall outside the accepted control variance. The FS wavelengths from the canopy dataset were classified using SVM and ND-SVM with the improvement shown in Table 2. The comparison for fixed leaves is shown by Tables 3 and 4.

It should be noted that whilst this method enhanced the rate of detection in this instance, it does not distinguish between disease and other stresses, not present in control plants, that may cause the reflectance to deviate beyond the devised limits. Hence, in practice, it could be used to detect areas of interest before a conventional SVM is applied to classify the type of stress; using a library of labelled responses. This is of particular interest due to the high classification rate from very early in the trial (09 DAI).

Although a larger population is required for phenotyping applications, an example of the difference in resistance of the four selected cultivars can be found in Table 4. Note that by tailoring the classifier to the correct type, the performance is improved significantly as the training sets become more representative with similar features and infection rates.

### 3D plant reconstruction

**Fig. 5:**
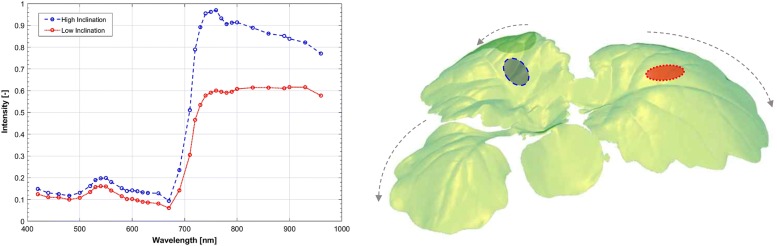
Orientation effects. Variation in reflectance measured at high (blue) [$50\,{^{\circ }}$] and low (red) [$10\,{^{\circ }}$] inclination normalisation (left) PS reconstruction of control oilseed rape plant, at 03 DAI with excessive curvature of leaves highlighted with arrows (right)

The effect of leaf orientation on the reflectance magnitude can be minimised using spectral pre-processing techniques [50], however, it still has a pronounced effect on the overall shape which can have adverse effects on the classification and thus increase the minimum detection time. This meant that for each plant there is detailed morphological information, which can give biomass and growth characteristics, and allow an insight into the variation of backscattered reflectance, see Fig. 5. This is important due to the growing interest in the apparent non-Lambertian reflectance properties of leaf-tissue at varying angles with respect to the imaging plane [51], which manifested in the lower classification levels on entire plant samples.

## Discussion

The classification accuracy of the plant analysis (83-97%) was in-line with existing literature (61–99%) [20] however, the time from inoculation to 90+% diagnosis was a significant improvement (12 DAI), particularly due to the clandestine nature of the hemibiotrophic pathogen at early stages of its life-cycle. The active MSI technique allowed detection of disease regions allowing for localised classification of infected tissue areas. The method, with application of machine learning algorithms, allowed infection detection for entire plants at 92% accuracy 13 days earlier than visual inspection. The authors would urge caution with the early rates of classification for ND-SVM, caused by the difference in residue left from the control and pathogen inoculum. Although this is of interest in this study, it is a similar infection mechanism that could occur in-field or during high-throughput trials, which did not give any visible symptoms and thus outlines the requirement for spectral applications.

The dual-scale nature of the experiment allowed for a detailed investigation into the orientation effects that are so problematical in the HSI imaging of plant material [51–53]. The detached-leaf assay also had a number of drawbacks despite being the measurement of reference for flat samples. This difference in the time-scales between the two experimental set-ups, was chosen to reduce the development of chlorosis on detached leaf assay but also meant the infection took longer to manifest; resulting in visual symptoms seven days later for detatched leaves. The simulated view of the entire leaf on a flat plane gave an important insight into the pathogen mechanism on a leaf scale (see Fig. 3) and gave an example of what could be seen on the canopy, should orientation reflections be fully compensated. Since the leaf images were taken alongside the canopy, the significance of this effect on detection rates can be examined (see Fig. 5) with an average increase in classification rate of 15% for flat orthogonal samples.

The 3D capabilities are still in the testing phase as there are a number of limitations to the current design. The PS method manages to represent the surface features with high-fidelity but suffers on discontinuous surfaces; to negate this an OSAVI mask is applied to remove non-plant shapes from the scan, but in future, the inclusion of structured light will prevent this and allow for more macro surface orientation based morphology to be extracted. The current LED design means that a Gaussian distortion is introduced into the reconstruction, which can be seen in the model in Fig. 5; as the curvature of the leaf tip is exaggerated.

The study found that automated FS can play an important role in improving classification performance, when compared to SVIs or even full spectra with SVM. This is due to the limitation of specific wavelengths with mixed relevance to disease ingress and additional data-points obfuscating the classification respectively. A comparison demonstrating the improvement is shown in Table 1. Another important finding was that the improvement in prediction accuracy between the conventional (SVM) and one-class (ND-SVM) classifiers, illustrated in Table 2.

There are not sufficient replicates of the cultivars to be able to infer a clear relationship between the resistance and classification rates. However, since these rates have been defined on visual symptoms [30] then they would not be a good reference when compared to destructive molecular techniques. This system would need some modification before it could be utilised outside the lab environment as the performance would be affected by additional stresses (e.g. nutrient deficiency) and ambient lighting; thus the current design may prove of more value in controlled indoor environments.

## Future work

There are a number of potential improvements to improve the data acquisition process, with a major focal point on fully incorporating the canopy orientation models into the spectral processing to remove the variation seen in Fig. 5. A faster global shutter system could replace the rolling shutter sensor; allowing a significant improvement in SNR. On-line visualisation of the multispectral cube and 3D information would inform the user of current limitations of imaging technique, which is often not found until the data is analysed. Finally, to improve the static nature of the current system, a common communication interface to a translational stage would allow integration in high-throughput applications. A revised system will be implemented in a field setting, incorporated into the spot-spraying equipment. This location has the added benefit of the shade created by the health and safety enclosures on all operational farm machinery, which in turn improves the SNR.

## Conclusions

In this paper, a low-cost active MSI system has been developed. Different analysis techniques have been used with the primary goal of evaluating the application of this system to the study of plant pathogen interactions. The findings clearly indicate the ability to detect disease using spectral information, with the minimum detection level affected by leaf orientation. The paper also exploits machine learning methods to extend the diagnosis beyond user supervised techniques. The outcome of this will not only help to detect the onset of disease but will also help in breeding varieties in the future by extending current breeding capabilities, by allowing for better differentiation of resistant cultivars.
